# Drive Force and Longitudinal Dynamics Estimation in Heavy-Duty Vehicles

**DOI:** 10.3390/s19163515

**Published:** 2019-08-11

**Authors:** Vicent Girbés, Daniel Hernández, Leopoldo Armesto, Juan F. Dols, Antonio Sala

**Affiliations:** 1 Instituto de Diseño y Fabricación (IDF), Universitat Politècnica de València, Camino de Vera s/n, 46022 Valencia, Spain; 2 Instituto de Automática e Informática Industrial (AI2), Universitat Politècnica de València, Camino de Vera s/n, 46022 Valencia, Spain

**Keywords:** sensor fusion, sampled-data, Kalman filter, dynamic systems, parameter identification, heavy vehicles, CAN bus, SAE J1939

## Abstract

Modelling the dynamic behaviour of heavy vehicles, such as buses or trucks, can be very useful for driving simulation and training, autonomous driving, crash analysis, etc. However, dynamic modelling of a vehicle is a difficult task because there are many subsystems and signals that affect its behaviour. In addition, it might be hard to combine data because available signals come at different rates, or even some samples might be missed due to disturbances or communication issues. In this paper, we propose a non-invasive data acquisition hardware/software setup to carry out several experiments with an urban bus, in order to collect data from one of the internal communication networks and other embedded systems. Subsequently, non-conventional sampling data fusion using a Kalman filter has been implemented to fuse data gathered from different sources, connected through a wireless network (the vehicle’s internal CAN bus messages, IMU, GPS, and other sensors placed in pedals). Our results show that the proposed combination of experimental data gathering and multi-rate filtering algorithm allows useful signal estimation for vehicle identification and modelling, even when data samples are missing.

## 1. Introduction

Nowadays, driving simulators are widely spread as an important tool in several areas like education or research. That is mostly due to the different advantages they offer compared to real vehicles [1]. Some useful features of simulators are the capability of stopping the simulation or giving feedback when used as a learning tool, the possibility of collecting massive amounts of data easily for driving analysis and the relatively low cost of the system compared to real vehicles, which saves money and time in the experimental process. In [2], under the so-called SAFEBUS project, experimental results are shown regarding some safety issues with urban buses, using both simulators and real experimental data. In addition, in [3,4], a simulated environment was used to evaluate a method to assist bus drivers through haptic devices.

**Figure 1:**
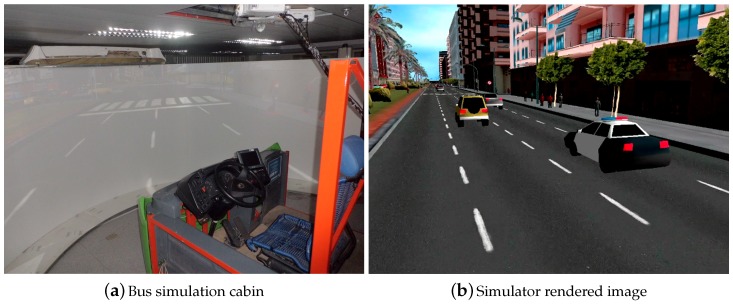
Bus simulation cabin and urban simulated environment.

However, one of the main inconveniences of using simulators is the well-known *simulator sickness* effect caused by a disconnection between what your eyes see and what your body feels. This problem has motivated this paper: in order to minimise this effect, we need to provide more realistic simulations. For this, we need to reproduce a good approximation of the vehicle kinematics/dynamics and feed that motion back to the simulation cabin, see Figure 1.

In recent years, naturalistic driving has arisen as one of the most advanced methods for road safety and driving behaviour studies [5,6], based on observing a representative group of drivers during a large amount of time, under real driving conditions; massive and reliable data are gathered for modeling human interaction with road environments [7]. However, in order to model longitudinal and lateral dynamic aspects of vehicles, specially designed closed-circuit experiments may be more appropriate to highlight the most relevant aspects of these dynamics [8].

Simulators must be validated so that drivers perceive a more realistic and immersive feeling and they can be compared to real driving. There are several studies on driving simulators validation, but the differences on uses and simulators make the validating process unique in each case [9]. Some common conclusions on driving simulators in different tests and contexts are that the validity between the real vehicle and the simulated one on non extreme speeds is good in terms of longitudinal control, and relatively good on lateral control [10,11]. Thus, in order to make a good simulator, the simulated vehicle must behave like the real vehicle in terms of linear and angular speed [12]. For instance, the authors of [13] validated the linear and lateral stability of an industrial vehicle using a simulator. On the other hand, the study carried out in [14] proved that a validated driving simulator can be used as a tool to assess traffic safety at signalized intersections. Another interesting point is that, in driving simulators with a large field of view, longitudinal speed can be estimated absolutely from environment information by the driver. Finally, controller tuning in mixed virtual and real environment also requires suitable identification and model validation steps [15].

### 1.1. Data Collection in Vehicles

In order to collect experimental data to model the vehicle for a simulator, specific tests designed to determine the different dynamics of the vehicle are proposed in [8]. For example, for the longitudinal dynamics, the described experiments include acceleration, braking and coast-down tests. Each one of these tests isolates one source of force applied to the vehicle, making the identification of their corresponding parameters much easier.

A Controller Area Network (CAN) in heavy-duty vehicles use J1939 protocol, as defined by the Society of Automotive Engineers (SAE) [16], which is used in vehicle networks for trucks and buses, agriculture and forestry machinery (ISO 11783), truck-trailer connections, diesel power-train applications, military vehicles (MiLCAN), fleet management systems (FMS), recreational vehicles, marine navigation systems (NMEA2000), etc. Due to technological limitations of some sensors, as well as CAN bus priority-based messaging policy, data are not generally available at fixed single sampling period. Thus, it is more realistic to assume that, in most cases, data might not be produced at the desired regular frequency for a good system identification (missing data problem), as it will be shown, later on, in our actual experimental datasets.

Different options are explored to record data during driving tests. The typical approach is using an Inertial Sensor Unit (IMU) with Global Positioning System (GPS) to estimate the position and speed of the vehicle [17,18]. However, a different option is possible: combining these IMU/GPS sensors with the low frequency internal information of the vehicle. This information is standardised, in case of heavy-duty vehicles, by the norm J1939 [16], where a CAN bus is used to communicate the different subsystems and Electronic Control Units (ECUs) of the vehicle. The authors of [19] proposed a commercial integrated circuit to connect to the CAN network, being able to read and write messages, record data and also to control the internal system. However, there are safer methods which use contactless magnetic devices to only register the CAN data, where the device is not physically connected to the network and, thus, cannot accidentally affect the control system of the vehicle. These safety aspects are key, for instance, in data collection from passenger buses which are the topic of this work. However, as a possible drawback, these contactless methods may lose connection for some time during the tests, due to electromagnetic disturbances or mechanical vibrations.

Many works have considered the use of vehicle’s internal network, both in cars and heavy-duty vehicles. For instance, an integrated self-diagnosis system was proposed in [20], where information from the sensors of an autonomous vehicle was collected, diagnosed, and reported to the driver using deep learning. A similar approach was done for monitoring and diagnostic of automobile smart and integrated control systems [21,22]. The authors of [23] participated in the DARPA Grand Challenge and, to control the vehicle, they had to access J1939 bus in order to measure vehicle speed, individual wheel speeds, engine revolutions per minute (RPM) and transmission gear status, and various temperature, pressure, and fluid level measurements. In [24], a modular controller for the IVECO ISG hybrid electric vehicle (HEV) was developed based on the J1939 CAN bus, whilst some authors implemented the SAE J1939 protocol to access the information of the distributed control system of electric city buses [25,26].

### 1.2. Contribution

**Figure 2:**
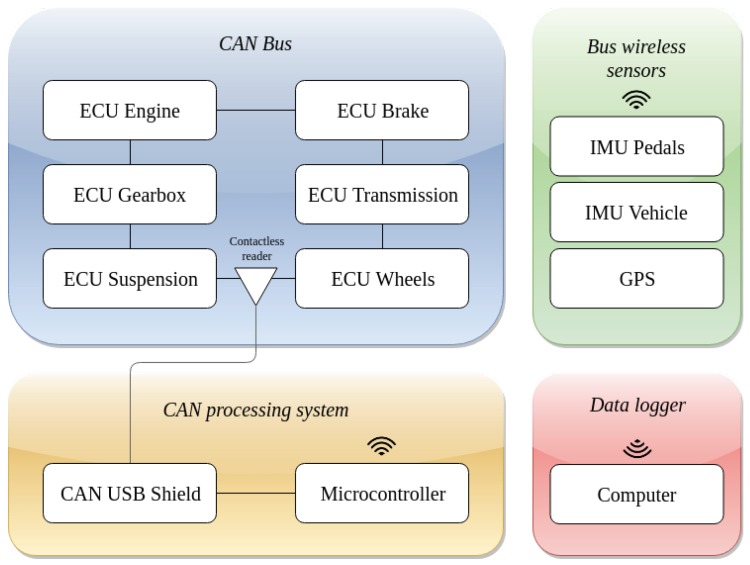
Diagram of the connections between the different data sources and the data logger. The external sensors communicate directly with the computer and the internal vehicle data are available from the CAN bus network employing a contactless reader. This data are then processed by a microcontroller and sent to the data logger.

To the authors’ knowledge, there is not prior literature work performing data fusion using J1939 bus, GPS and IMU data, with the main purpose of enhancing irregularly-sampled data for system identification. In this sense, this paper deals with data sensor fusion techniques of systems with multi-rate data coming from industrial communications, such as CAN bus, combined with measurements from several sensors such as GPS and several IMUs, as shown in Figure 2. The aim is to estimate pedals position, velocity, acceleration and engine torque. The potential advantage of this fusion is that, while traditional fusion of IMU and GPS provide reasonable estimations, consideration of CAN data will improve the estimation, particularly in estimating vehicle’s velocity at low speeds [27]. In addition, at faster speeds, it will also help, particularly in common scenarios where the velocity is basically constant and, henceforth, the actual acceleration is within the order of magnitude of acceleration noise. In this sense, a Kalman Filter (KF) is the optimal filtering solution for linear stochastic dynamic systems under common Gaussian assumptions.

The main contributions of the paper are: a non-invasive data acquisition hardware/software setup to carry out several experiments with an urban bus, in order to collect data a vehicle’s CAN network and other embedded systems (IMU, GPS, and other sensors placed in pedals and steering wheel); as well as a non-conventional sampling data fusion using a multi-rate KF to fuse data gathered from different sources. Our results show that the proposed estimated signals can be used for vehicle dynamics identification and modelling, even when data samples are missing. The ultimate goals would be to identify a dynamic model from data gathered from a real bus to reproduce such dynamics on a bus simulator, as shown in Figure 1. Possible uses of these ideas in complex environments, such as testing autonomous algorithms in safety applications, training professional bus drivers on artificial scenarios or collision avoidance algorithms, are out of the scope of this paper.

The paper is organised as follows. Section 2 introduces some preliminaries about sampled-data systems and sensor fusion, as well as basic concepts about the CAN communication protocol SAE J1939 for heavy-duty vehicles. The problem statement is explained in Section 3. The proposed materials and methods used during the experimentation are defined in Section 4. Section 5 shows the main results obtained from driving tests carried out to evaluate the proposed methodology. Finally, the paper ends with a discussion and a summary of the main contribution of the paper in Section 6.

## 2. Preliminaries

### 2.1. Multi-Rate Sensor Fusion

Multi-rate sensor fusion techniques for sampled-data systems deal with dynamic systems with inputs and outputs working at different sampling rates [28,29,30]. These techniques arise from the fact that, in many real systems, sensors provide data at different sampling periods due to the nature of their sensing technology. Similarly, actuators in control applications might work at a given maximum frequency due to technological aspects. It is well known that control systems are significantly affected by the sampling period.

There are situations in which we can assume that there is a periodicity between all sampling rates and, as a consequence, we can work with periodic sampled-data systems or, at least, work with a *base* sampling period which represents the least common multiple (LCM) of all sampling rates.

Let *x* be the state of a discrete-time stochastic linear dynamic system; $\epsilon_{w}$ and $\epsilon_{v}$ independent Gaussian noises, known as process noise and measurement noise, respectively; and *y* the measurement vector described as follows:

(1) $$\begin{array}{cc} x_{+} & =Ax+B\epsilon_{w}, \end{array}$$

(2) $$\begin{array}{cc} y & =Cx+\epsilon_{v}, \end{array}$$

where *A*, *B* and *C* are the system dynamics matrix, noise coupling matrix and output measurement matrix, respectively. Gaussian noises have zero mean and covariance *Q* and *R*, respectively, i.e., $E(\epsilon_{w})=0$, $E(\epsilon_{v})=0$, $E(\epsilon_{w}\epsilon_{w}^{T})=Q$ and $E(\epsilon_{v}\epsilon_{v}^{T})=R$ and $E(\epsilon_{w}\epsilon_{v}^{T})=0$.

A multi-rate Kalman filter can be implemented as a standard KF with conditioned update equations based on the availability of data [29]. Thus, depending on the available sensors, the output Equation (2) varies both the size and contents of *C* and $\epsilon_{v}$. As a result, the multi-rate Kalman filter ends up being implemented as the following iterations executed at the fast base period:

(3) $$\begin{array}{cc} {\hat{x}}_{+} & =A\hat{x}, \end{array}$$

(4) $$\begin{array}{cc} P_{+} & =APA^{T}+BQB^{T}, \end{array}$$

(5) $$\begin{array}{cc} K_{s} & =PC_{s}^{T}{\left(C_{s}P_{+}C_{s}^{T}+R_{s}\right)}^{-1}, \end{array}$$

(6) $$\begin{array}{cc} {\hat{x}}_{+} & ={\hat{x}}_{+}+K_{s}\left(y_{s}-C_{s}x_{+}\right), \end{array}$$

(7) $$\begin{array}{cc} P_{+} & =\left(I-KC_{s}\right)P_{+}, \end{array}$$

where sub-index *s* denotes *variable-size* matrices and vectors, depending on the actual vector of available samples, $y_{s}$, at the moment the computations are executed. Note that, if there were no samples at a particular time instant, Equations (5)–(7) should not be executed, so uncertainty propagation would be *open-loop* in such a case.

### 2.2. SAE J1939 CAN Protocol for Heavy Vehicles

The Controller Area Network is a serial bus protocol, widely used in the industry and, especially, in the automotive sector. It works in various electrical environments without any issues, allowing communication between controllers, actuators and sensors. Its simplicity is among the main advantages, as it uses a single twisted pair wire, terminated with a resistor at each end, with nodes connected to it. CAN bus reduces wiring, since it is a distributed control architecture and this ensures enhancing of the overall performance because the system is robust towards failure of subsystems and electromagnetic interference.

The broadcasting of messages is based on a producer–consumer principle. One node, when sending a message, will be the producer while all other nodes are the consumers. All nodes in a CAN network receive the same message at the same time [31]. Messages in CAN are not confirmed because that would unnecessarily increase the bus traffic. However, as it is a multi-master protocol, it is likely to have undesirable interactions between nodes. These interactions are produced when two or more ECUs try to get access to the network simultaneously. In this case, arbitration determines which node takes priority. CAN messages are prioritised via IDs, so that the highest priority IDs are non-interrupted [31]. Therefore, in the arbitration, the “winner” continues sending (without delay), while the other nodes need to wait during the data transmission.

The SAE J1939 protocol is one of the most popular higher layer protocols based on a CAN bus. Thus, it provides serial data communications between distributed microprocessor systems or ECUs, without complex dedicated wiring in between, which simplifies the vehicle’s internal network [16]. It is currently used by diesel-powered applications, including vehicles, engines and some industrial purposes because it is the recommended standard for diagnosis and communication among the different ECUs of vehicles via CAN bus. However, it is especially important in the field of heavy-duty vehicles, where many well-known manufacturers of trucks and buses already implement this norm.

The SAE J1939 message format consists mainly of two different fields, the identification (ID) field and the data field. The ID field is preceded by the three bits that control the message priority during the arbitration process. The identification of the message has 18 bits and defines the Parameter Group Number (PGN), which identifies the message type. The other fields of the message are its source address, the control field, the cyclic redundancy check field and the acknowledgement and end of frame bits.

**Figure 3:**
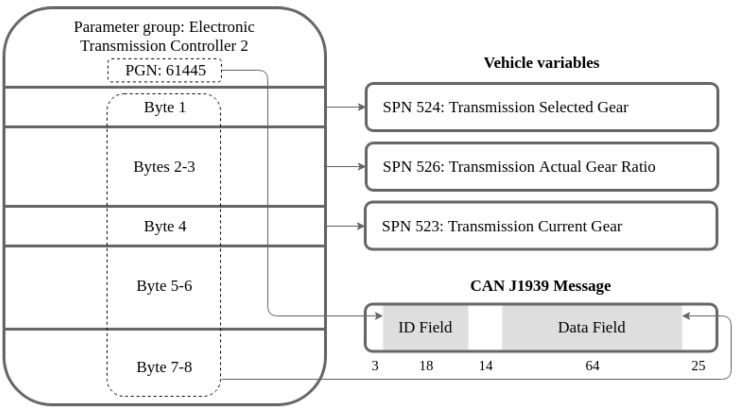
J1939 PGN 61443 message, which contains actual gear ratio, selected gear and current gear.

Some examples of PGNs are the Cruise Control/Vehicle Speed, Electronic Transmission Controller or Vehicle Dynamic Stability Control. Each group contains different information in the data field, classified by Suspect Parameter Numbers (SPN). Every message implements a different structure to fill its corresponding variables within the 64 bit data field, although not every data byte is always used. For instance, Figure 3 shows the full structure of the message Electronic Transmission Controller 2 (ETC2 with PGN 61445) that contains the actual gear ratio and both selected and current gears. A complete list of the used messages and variables for this project is available in Appendix A.

In this protocol, each message has its own range of transmission rates, but the priority of each group may change and affect that rate. Depending on the level of priority, transmission rates vary from 10 ms, for high-priority nodes, to 1 s, for low-priority nodes. Messages with higher priority will gain bus access within the shortest time, even when the bus load is high due to the number of lower priority messages. A value of 0 (000b) represents the highest priority, assigned to time-critical messages, such as torque control data from transmission to engine. A value of 8 (111b) represents the lowest priority, suitable for, say, some configuration data.

## 3. Problem Statement

In order to provide suitable and valid data for dynamic modelling of vehicles, we need to develop appropriate methods for estimation and filtering of vehicle-related signals. Therefore, in order to estimate variables related to vehicle’s longitudinal movement, we need to measure engine torque/RPM, clutch position, gear ratios, pedals position, and brake pressure, among others. This usually requires considering data coming from sensors located at very specific places, which might be difficult to install for non-manufacturers. This paper focuses on longitudinal dynamics; actually, the vehicle’s lateral dynamic estimation would require a different specific experimental setup, but the main ideas of this paper can be applied to it.

In addition, mounting external devices in a vehicle would require homologation in order to comply with the regulations, since the original structure of the vehicle would be modified. As representative examples, if we need to measure pedals position, we can install a potentiometer on the pedal axis, or we might need a load cell directly attached to the drive axis if we need to measure engine torque. This setup is clearly costly, difficult to achieve in many practical situations, and it might vary from one vehicle to another. Thus, we are seeking a non-invasive setup that will allow us to perform experiments, even at urban scenarios, without affecting normal driving, in order to collect data under different operating conditions.

As previously mentioned, heavy-duty vehicles implementing SAE J1939 provide some of the required data. However, due to the nature of broadcasting, some low-priority CAN messages come asynchronously [16] and, generally, at a low frequency, which is acceptable for monitoring and diagnosis purposes, but not for dynamic parameters’ identification. Furthermore, we need to include additional sensor sources, such as IMUs and GPS in a non-invasive setup, in order to overcome some of the limitations of contactless CAN sniffers that might miss some messages due to, for instance, electromagnetic disturbances or relative motion. Thus, under such scenario, it is necessary to assume that data comes sparsely and at different sampling rates, and suitable sensor fusion techniques for later dynamic model identification should be devised.

## 4. Materials and Methods

### 4.1. Materials

The urban bus used for the experimentation was a MAN 14250 HOCL-NL with the following specifications: mass m= 11,750 kg, length L= 12,000 mm, width W=2550 mm, overall height H=3160 mm, distance between axes $D_{A}=5875$ mm, rear overhang $O_{R}=3415$ mm, front track $T_{F}=2086$ mm and rear track $T_{R}=1826$ mm.

**Figure 4:**
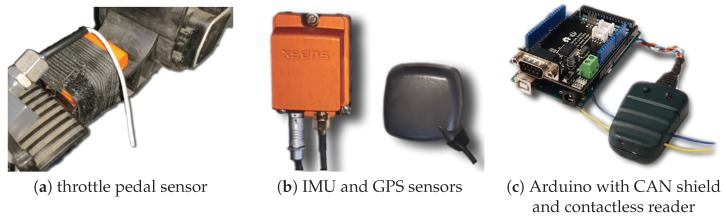
Sensors used for data acquisition and logging.

Figure 4 shows sensors and other electronic devices used for the data acquisition system. As inertial measurement units, three wireless motion trackers (Xsens MTw Awinda, Enschede, The Netherlands) were placed in throttle and brake pedals, as depicted in Figure 4a. Each tracker is a full IMU with the following components: three-axis magnetometer (full scale ±1.9 Gauss), three-axis gyroscope (full scale ±34.9rad/s) and three-axis accelerometer (full scale $\pm 160\;{m/s}^{2}$). The dynamic accuracy of the orientation is 0.013 rad for roll/pitch angles and 0.026 rad for yaw angle, where both values are the quadratic mean, also known as root mean square (RMS).

To track the position of the bus and other kinematic and dynamic variables, an Xsens MTi-G-710 GNSS inertial measurement unit with GPS was mounted on the vehicle’s centre of rotation (see Figure 4b). The IMU incorporates the following components: three-axis magnetometer (full range ±8 Gauss, RMS noise 0.5mG), three-axis gyroscope (full range ±7.85rad/s, bias error 0.0035rad/s), three-axis accelerometer (full range $\pm 200\;{m/s}^{2}$, bias error $0.05\;{m/s}^{2}$) and barometer (full range 30–110 kPa, RMS noise 3.6Pa). The dynamic accuracy of the orientation is 0.005 (pitch/roll) and 0.014 (yaw). Regarding the GPS, the horizontal accuracy is 1 m (Cartesian coordinates x/y) and the vertical accuracy is 2 m (z coordinate).

A contactless CAN connector was used to safely read data from vehicle CAN bus (see Figure 4c). Using this device, data reading is non-invasive as it occurs without electrical connection and without damaging CAN wires. It works in “listen” mode only, i.e., it does not change original J1939 messages and does not send any signals to the CAN bus. This CAN device was connected to a micro-controller Arduino Mega 2560 with a CAN-BUS shield, which transmitted all collected data to a Raspberry PI 3 Model B+ with an embedded Linux (used as the main computer for data logging).

### 4.2. Method

In this paper, we propose using a multi-rate sensor fusion algorithm considering the multi-rate nature of data. In particular, we use a multi-rate Kalman Filter that performs the prediction and size-varying update, considering only available data. The chosen option to deal with a multi-rate setup is considering a base period to be the maximum common divisor of all underlying sampling periods in a particular problem [28]. In our case, we have chosen T=0.01 s as the base period, and checked that all measurements in our datasets come at an (irregularly spaced) multiple of such a period.

In the sequel, super-indices br and th correspond to variables related to brake and throttle pedal, respectively. In addition to this, super-index GPS corresponds to data coming from GPS sensor (Figure 4b); super-index IMU refers to IMU sensors (Figure 4a,b, each one for the corresponding signal to estimate); and super-index CAN corresponds to all available measurements from CAN J1939 bus (Figure 4c). In particular, we use PGNs 61441, 61443, 61449, 65132, 65215 and 65265 of this protocol (see details in Appendix A). As an example, $v^{CAN}$ will denote linear velocity coming from the CAN bus (obtained from the tachograph and the wheels’ velocities), whereas $s^{GPS}$ will refer to linear displacement read from the GPS, obtained from raw differential GPS data over the Cartesian space (details omitted for brevity).

Dynamic models and signals used to estimate the position of the pedals are introduced next. Estimation of each variable of interest (pedals’ position) is implemented independently, but their state vector, system dynamics, measurement equations, etc. follow the same structure. The position is described by a parameter ρ, which is the variable of interest, with a super-index to refer to each independent variable. For both pedals, $\rho^{br}$ and $\rho^{th}$ represent the angular position with respect to the resting state.

Although not strictly necessary, the position of both pedals is usually normalised to the range [0,100]%. After a proper calibration is done, values can be converted easily from angle to percentage and vice versa. In our experimentation, the equations used for such conversion are the following: $\rho_{\%}^{br}=238.095\cdot \rho^{br}+157.143$ for the brake, and $\rho_{\%}^{th}=-270.271\cdot \rho^{th}+43.243$ for the throttle pedals.

IMUs can be affected by bias due to calibration errors and other external effects, such as magnetic field perturbations. Thus, in the estimation, we have included an offset because there is an unmeasured drift produced by the integration of gyroscope and accelerometer’s biases. Hence, the state vector is expressed as $x^{*}={\left[\rho^{*}\;\rho_{o}^{*}\right]}^{T}$, where * can be any super-index depending on the system/signal they represent (br, th). The output measurement vector is $y^{*}={\left[\rho^{*,IMU}\;\rho^{*,CAN}\right]}^{T}$, which includes pedal position measured by their respective IMU, or the value obtained from CAN bus for each signal. We assume that process noise vector is $\epsilon_{w}^{*}={\left[\epsilon_{\rho^{*}}\;\epsilon_{\rho_{d}^{*}}\right]}^{T}$; measurement noise vector is $\epsilon_{v}^{*}={\left[\epsilon_{\rho^{*}}^{IMU}\;\epsilon_{\rho^{*}}^{CAN}\right]}^{T}$; and system dynamics are described as:

(8) $$x_{+}^{*}:=\left[\begin{array}{cc} e^{-B_{w}^{*}T} & 0\\ 0 & 1 \end{array}\right]\left[\begin{array}{c} \rho \\ \rho_{o} \end{array}\right]+\left[\begin{array}{cc} 1-e^{-B_{w}^{*}T} & 0\\ 0 & T \end{array}\right]\left[\begin{array}{c} \epsilon_{\rho^{*}}\\ \epsilon_{\rho_{d}^{*}} \end{array}\right]\;y^{*}:=\left[\begin{array}{cc} 1 & 1\\ 1 & 0 \end{array}\right]\left[\begin{array}{c} \rho^{*}\\ \rho_{o}^{*} \end{array}\right]+\left[\begin{array}{c} \epsilon_{\rho^{*}}^{IMU}\\ \epsilon_{\rho^{*}}^{CAN} \end{array}\right],$$

where $B_{w}$ is the assumed bandwidth of the “ground-truth” signal we are trying to estimate, in rad/s, using the exponential formula to compute its associated dynamics in a first-order discrete-time setup at the base period *T*. In the experimentation, we used $B_{w}^{br}=B_{w}^{th}=0.1$ Hz.

On the other hand, in order to estimate the vehicle’s longitudinal kinematics, the state vector includes travelled distance *s*, linear velocity *v* and linear acceleration *a*. In order to compensate offsets, in the state vector, we also need to include biases for the velocity and acceleration due to the nature of IMU measurements, as well as non-systematic errors of the wheel’s speed measurements from CAN data (such as incorrect tyre pressure, wheels misalignment, etc.). Thus, the state vector for vehicle’s longitudinal kinematics is defined as $x={\left[s\;v\;a\;a_{o}\;v_{o}\right]}^{T}$, $a_{o}$ and $v_{o}$ being the offsets of the acceleration and velocity, respectively. The output vector includes data from GPS, IMU and CAN as follows: $y={\left[s^{GPS}a^{IMU}\;v^{CAN}\;a^{CAN}\right]}^{T}$; process noise vector is assumed to be $\epsilon_{w}={\left[\epsilon_{s}\;\epsilon_{a}\;\epsilon_{a_{o}}^{IMU}\;\epsilon_{v_{o}}^{CAN}\right]}^{T}$; measurement noise vector is $\epsilon_{v}={\left[\epsilon_{s}^{GPS}\;\epsilon_{a}^{IMU}\;\epsilon_{v}^{CAN}\;\epsilon_{a}^{CAN}\right]}^{T}$; and system dynamics and output measurement equation are described as:

(9) $$\begin{array}{cc} x_{+} & =\left[\begin{array}{ccccc} 1 & T & 0 & 0 & 0\\ 0 & 1 & T & 0 & 0\\ 0 & 0 & 1 & 0 & 0\\ 0 & 0 & 0 & 1 & 0\\ 0 & 0 & 0 & 0 & 1 \end{array}\right]\left[\begin{array}{c} s\\ v\\ a\\ a_{o}\\ v_{o} \end{array}\right]+\left[\begin{array}{cccc} \frac{1}{2}T^{2} & 0 & 0 & 0\\ 0 & 0 & 0 & 0\\ 0 & T & 0 & 0\\ 0 & 0 & T & 0\\ 0 & 0 & 0 & T \end{array}\right]\left[\begin{array}{c} \epsilon_{s}\\ \epsilon_{a}\\ \epsilon_{a_{o}}^{IMU}\\ \epsilon_{v_{o}}^{CAN} \end{array}\right] \end{array}$$

(10) $$\begin{array}{cc} y & =\left[\begin{array}{ccccc} 1 & 0 & 0 & 0 & 0\\ 0 & 0 & 1 & 1 & 0\\ 0 & 1 & 0 & 0 & 1\\ 0 & 0 & 1 & 1 & 0 \end{array}\right]\left[\begin{array}{c} s\\ v\\ a\\ a_{o}\\ v_{o} \end{array}\right]+\left[\begin{array}{c} \epsilon_{s}^{GPS}\\ \epsilon_{a}^{IMU}\\ \epsilon_{v}^{CAN}\\ \epsilon_{a}^{CAN} \end{array}\right]. \end{array}$$

**Figure 5:**
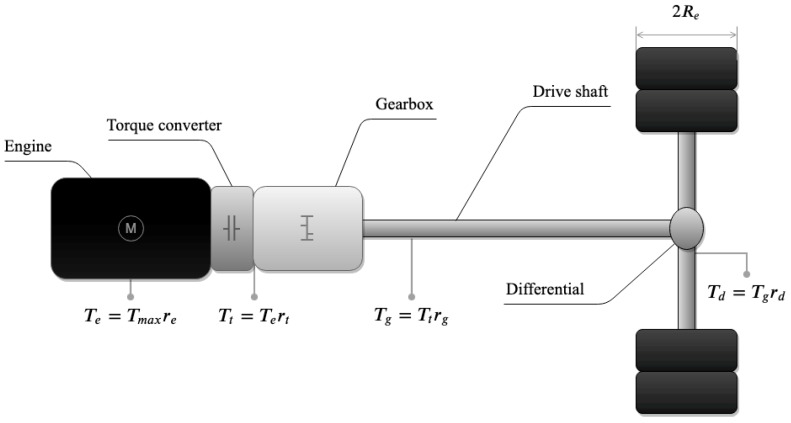
Powertrain diagram.

Finally, to show how the proposed method can be used for vehicle dynamics identification, we propose to estimate the drive force produced by the engine at a high sampling rate. This estimation will be obtained as a consequence of measuring CAN messages (with PGNs 61443, 61444, 61445) with information from the vehicle’s powertrain (i.e., engine, clutch, gearbox, drive shaft, differential, etc.), as shown in Figure 5. Hence, the traction force $F^{CAN}$ produced by the drive engine can be computed as

(11) $$F^{CAN}=\frac{T_{max}r_{e}^{CAN}r_{t}^{CAN}r_{g}^{CAN}r_{d}^{CAN}}{R_{e}},$$

where $T_{max}$ is an engine’s maximum torque; $r_{e}^{CAN}$ is engine’s load, which depends on throttle pedal position and current velocity, among other things; $r_{t}^{CAN}$ is the transmission ratio of the torque converter; $r_{g}^{CAN}$ is the transmission gear ratio, which depends on the engaged gear; $r_{d}^{CAN}$ final-drive gear ratio of the differential; and $R_{e}=\mu R$ is the effective tire radius, with *R* the maximum wheel radius and μ the reduction factor produced by vehicle’s weight and tire deformation.

In addition, vehicle acceleration (to be estimated with the vehicle kinematic multi-rate Kalman filter (9)–(10)) will be used to approximately compute the net drive force, $F_{n}$, as follows:

(12) $$F_{n}=a\cdot m\cdot f_{m},$$

with $f_{m}$ being the mass factor that approximates the equivalent mass of vehicle’s rotating parts and, according to [8], can be computed easily as $f_{m}=1.04+0.0025{\left(r_{g}^{i}r_{d}\right)}^{2}$, with $r_{g}^{i}$ the gear ratio of the *i*-th gear.

There are some remaining unmodeled forces, denoted as $F_{o}$, which represent a combination of losses due to air resistance $F_{a}$ (aerodynamic drag force), rolling resistance $F_{r}$ (produced by friction between surface and wheels), and gradient resistance $F_{g}$ (produced by the effect of the vehicle’s weight in slopes), so that $F_{o}\approx F_{a}+F_{r}+F_{g}$. The sum of all these unmodeled losses will be indirectly estimated, by our sensor fusion setup, as the discrepancy between the engine’s drive force, *F*, and the net drive force computed in Equation (12), i.e., $F_{o}=F-F_{n}$.

Hence, the state vector is expressed as $x={\left[F_{n}\;F_{o}\right]}^{T}$, where $F_{n}$ is the net drive force to estimate and $F_{o}$ are the losses due to the mechanical drive transmission. The output measurement vector is $y^{*}={\left[F^{CAN}\;{\hat{F}}_{n}^{a}\right]}^{T}$, which includes the drive force obtained from CAN, as well as the estimated drive force (without losses from the vehicle dynamic model) obtained from the estimated acceleration of the model (9) inserted into Equation (12), i.e., ${\hat{F}}_{n}^{a}:=\hat{a}\cdot m\cdot f_{m}$. We assume that process noise vector is $\epsilon_{w}={\left[\epsilon_{F_{n}}\;\epsilon_{F_{o}}\right]}^{T}$; measurement noise vector is $\epsilon_{v}^{*}={\left[\epsilon_{F}^{CAN}\;\epsilon_{{\hat{F}}_{n}}^{a}\right]}^{T}$. In this case, the system dynamics is described as:

(13) $$x_{+}:=\left[\begin{array}{cc} e^{-B_{w}^{*}T} & 0\\ 0 & 1 \end{array}\right]\left[\begin{array}{c} F_{n}\\ F_{o} \end{array}\right]+\left[\begin{array}{cc} 1-e^{-B_{w}^{*}T} & 0\\ 0 & T \end{array}\right]\left[\begin{array}{c} \epsilon_{F_{n}}\\ \epsilon_{F_{o}} \end{array}\right]\;y:=\left[\begin{array}{cc} 1 & 1\\ 1 & 0 \end{array}\right]\left[\begin{array}{c} F_{n}\\ F_{o} \end{array}\right]+\left[\begin{array}{c} \epsilon_{F}^{CAN}\\ \epsilon_{{\hat{F}}_{n}}^{a} \end{array}\right].$$

### 4.3. Data Collection from Driving Experiments

Several driving tests were carried out in order to collect data. In this paper, we focused on straight driving tests in order to estimate the drive force of the motor and also to get data for later identification of other bus elements, relevant for driving simulator validation, currently under research. All considered tests here start with the bus completely stopped. Then, the driver presses the throttle pedal and the vehicle speeds up until a certain linear velocity is reached. Finally, the driver releases the throttle and presses the brake until the bus is stopped. The experiment was repeated at different velocities (20 km/h, 40 km/h and 60 km/h) and with different levels of braking (low, medium and high).

**Figure 6:**
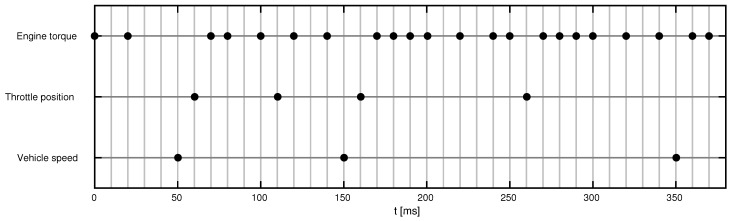
Timestamps for some representative CAN variables in one of the driving tests.

After gathering all CAN bus messages, we faced some issues when trying to process the data to estimate some signals at high sampling rates. Even though the CAN bus is synchronous, based on the fact that SAE J1939 is a priority-based protocol with many ECUs trying to publish their data through the network, some low-priority messages might come unevenly due to bus overload. In this sense, we found that, for some variables, there were gaps without any data. Time values for some representative CAN variables are shown in Figure 6. The percent engine torque has the highest default priority and has a variable transmission rate dependent on the engine speed, reaching the fastest transmission average rate of 100 Hz. The percent throttle position has an average rate of 20 Hz and the tachometer measured velocity has an average rate of 10 Hz. These two lower priority variables often come asynchronously due to large jitter.

In some cases, the time distance between samples is long (for instance, 200 ms between two speed measurements in Figure 6, bottom line), so linear interpolation between samples and other similar naive approaches would provide a too coarse estimate. Multi-rate KF sensor fusion significantly improves such estimation, as it is more robust against missing data and unmodeled measurement noises. Finally, it is important to remark that the proposed algorithm is designed for data coming synchronously at the base period; if timestamps do not coincide with such period, rounding to the nearest multiple of the sampling time would be recommended and, actually, for computational/storage reasons, our hardware and software setup did it accordingly. Intermediate sampling times are rounded to the nearest multiple of the sampling period. As the base period is very small compared to the signal bandwidths, the jitter arising from this rounding is negligible.

## 5. Results

For brevity, among all tests, only the results of the straight driving test at 60 km/h and medium braking are used. Considering the measurement uncertainties, covariance matrices are initialised as follows: $P_{0}^{br}=P_{0}^{th}=\mathrm{diag}\left(\left\{1,5^{2}\right\}\right)$, with the following process noise matrix $Q^{br}=Q^{th}=\mathrm{diag}\left(\left\{10000^{2},1^{2}\right\}\right)$ and with the sensor noise matrix $R^{br}=R^{th}=\mathrm{diag}\left(\left\{0.1^{2},0.1^{2}\right\}\right)$ for the pedals.

On the other hand, for vehicle’s kinematics estimation (Here, we use super-index vk to denote vehicle’s kinematics.), initial covariance matrices are set to $P_{0}^{vk}=\mathrm{diag}\left(\left\{5^{2},0.2^{2},0.1^{2},0.01^{2},0.01^{2}\right\}\right)$, with the following process noise matrix $Q^{vk}=\mathrm{diag}\left(\left\{50^{2},2^{2},0.1^{2},0.01^{2}\right\}\right)$ and sensor noise matrix $R^{vk}=\mathrm{diag}\left(\left\{4^{2},0.02^{2},0.5^{2},2^{2}\right\}\right)$.

Finally, the state covariance matrix for drive force estimation is initialised as follows: $P_{0}^{df}=\mathrm{diag}\left(\left\{1^{2},1^{2}\right\}\right)$, with the following process noise matrix $Q^{df}=\mathrm{diag}\left(\left\{100^{2},1^{2}\right\}\right)$ and with the sensor noise matrix $R^{df}=\mathrm{diag}\left(\left\{0.1^{2},1^{2}\right\}\right)$.

**Figure 7:**
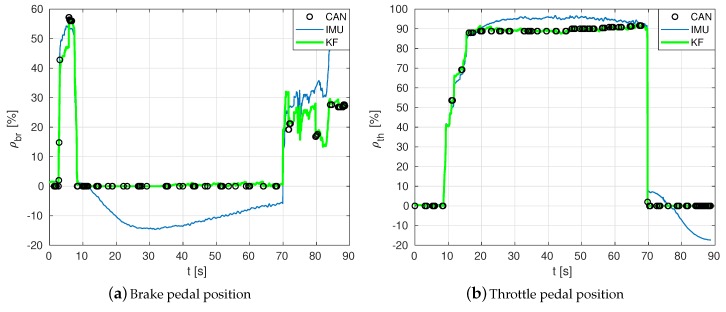
Estimation at a high rate of the positions of brake and throttle pedals (all in %).

**Figure 8:**
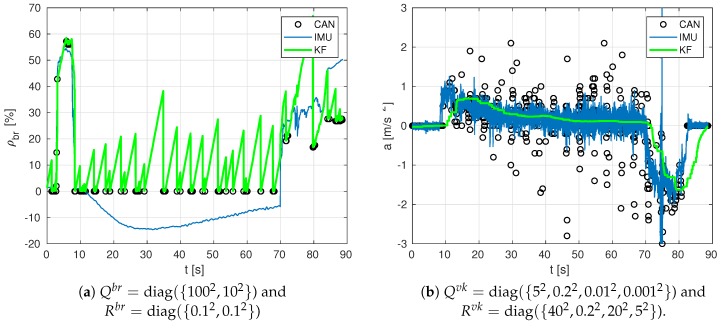
Estimation at a high rate of the position of brake pedal (in %) and vehicle’s acceleration, for the test at 60 km/h and high braking, with two examples of incorrect covariance matrices adjustment.

**Figure 9:**
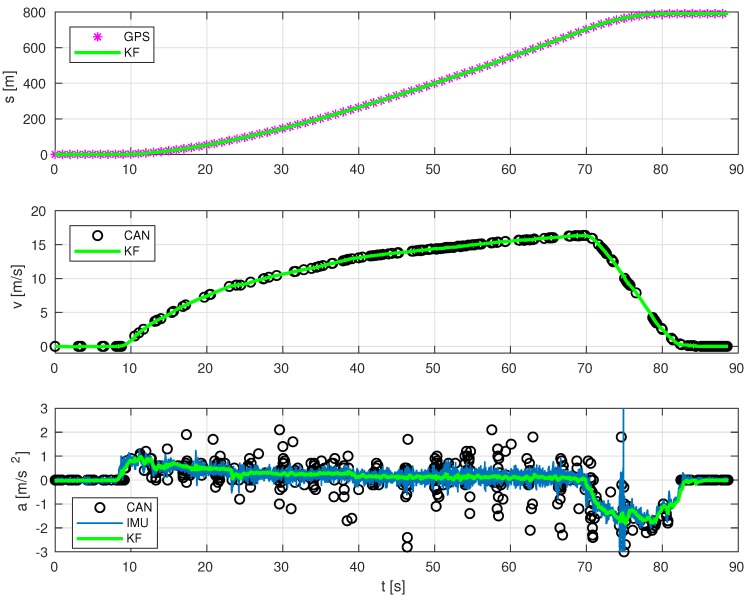
Estimation at a high rate of vehicle’s position, velocity and acceleration.

For a deeper understanding, Figure 8a shows the estimation of the brake pedal corresponding to the experiment of Figure 7a with an intentional choice of incorrect covariance parameters, which highlights the relevance of tuning these parameters in practical applications. Figure 8b also shows the relevance of correct choices of covariance parameters in acceleration estimates: the incorrect results in the figure can be compared against the correct ones in the bottom plot of Figure 9. As a consequence, the proposed values at the beginning of the section have been carefully selected so that they work properly in all available experiments.

Given the overall focus on driving simulation of our research, we have tailored our sensor-fusion data processing towards estimating: (a) pedal positions (driver inputs), (b) longitudinal kinematics (the actual position, speed and accelerations that the driving simulator must replicate in response to the said inputs), and (c) engine drive force (in order to accurately simulate the engine, clutch and gear behaviour).

**Pedal position estimation results.**Figure 7 shows some results on estimating the position, in percentage, of brake pedal $\rho^{br}$ and throttle pedal $\rho^{th}$. Estimations at a high rate of longitudinal kinematics can be seen in Figure 9. The multi-rate Kalman Filter algorithm fuses data from several sources coming at different sampling rates: IMU (acceleration), GPS (displacement position) and CAN network messages (velocity and acceleration, obtained from PGNs 65132, 61449, 65215 and 65265).

**GPS+IMU+CAN fusion in longitudinal kinematics estimation.** As observed in Figure 9, the estimated position has no bias, as expected, because GPS measures do not have it either. However, since it is a high-rate estimation, information is updated at 100 Hz, instead of the common operation rate of such sensors, which was 1 Hz in our experimental setup. 

The only actual measurement of velocity comes from vehicle’s internal odometry, since the other two possible sources would be integration of acceleration coming from IMU (blue crosses) and derivation of travelled distance computed from GPS position (magenta asterisks). However, the former can have a high bias after a while, whilst the latter is very noisy due to inaccurate and jumpy readings from satellites.

It is well known that odometry based only on encoders or tachographs is not reliable, as its accuracy depends on many factors, such as wheel radius (which depends on tire pressure, as they can be more or less inflated, as well as actual bus load) or the wheel’s slippage (which vary in different surfaces), among other things. However, although wheel velocity and linear acceleration coming from the CAN network might have an offset at certain speed (due to wheels’ misalignment, different wheels pressure, or discrepancies between measures of tachometers or encoders), estimation errors of vehicle’s linear velocity can be reduced thanks to the information given by CAN bus, even when it comes very sparse and at different rates (not shown for brevity). For instance, in the time range t∈[83,89] s in Figure 9, GPS was outputting non-constant positions originating a velocity offset. The same happens to the IMU that returns a certain value of acceleration producing non-zero linear velocity. However, the estimation of linear velocity is improved by fusing these signals with velocity readings from the CAN bus, which has zero offset when the vehicle is stopped.

Based on the results obtained from the experimentation, we identified the average offset values of the data coming from the CAN network (velocity offset $v_{o}^{CAN}$ was not statistically different from zero) and signals from the IMU sensor (accelerometer offset $a_{o}^{IMU}=-0.004143\;{m/s}^{2}$). These offset values were validated using data from other driving tests described in Section 4.3 showing that they laid in the ±3σ confidence interval.

**Figure 10:**
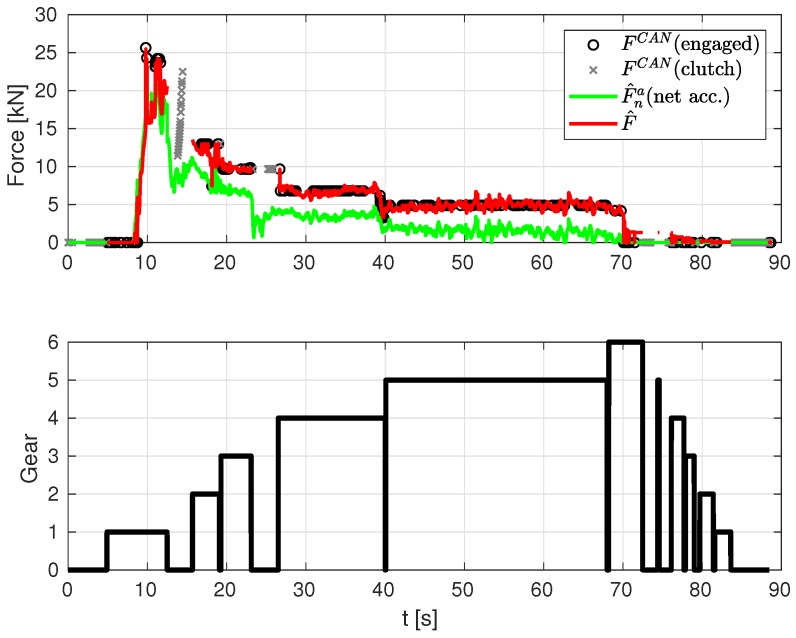
Engine force from CAN messages (with gear engaged and during shift) and estimated force (net acceleration) and estimated force with losses estimation.

**Drive-train engine force estimation.** Finally, Figure 10 depicts: the estimated drive net force ${\hat{F}}_{n}^{a}$ without considering losses (green line) obtained from the estimated acceleration inserted into Equation (12); the estimated drive force considering losses (red line) obtained from sum of the two estimated states in Equation (13); and, lastly, the engine drive force measurements obtained from the CAN bus (black circles). As can be seen, the estimated drive force (red line) is close to the one obtained from CAN, which validates the method proposed in this paper. Note that the data segments where the clutch was active and no effective gear was engaged are not considered in this filter because the engine is “disconnected” from the powertrain (clutch status was retrieved from CAN bus signals).

### Robustness Analysis

The collection of available driving tests has been used to analyse the robustness of the proposed sensor fusion setting. Let us now describe some of the different considered aspects for such analysis.

**Figure 11:**
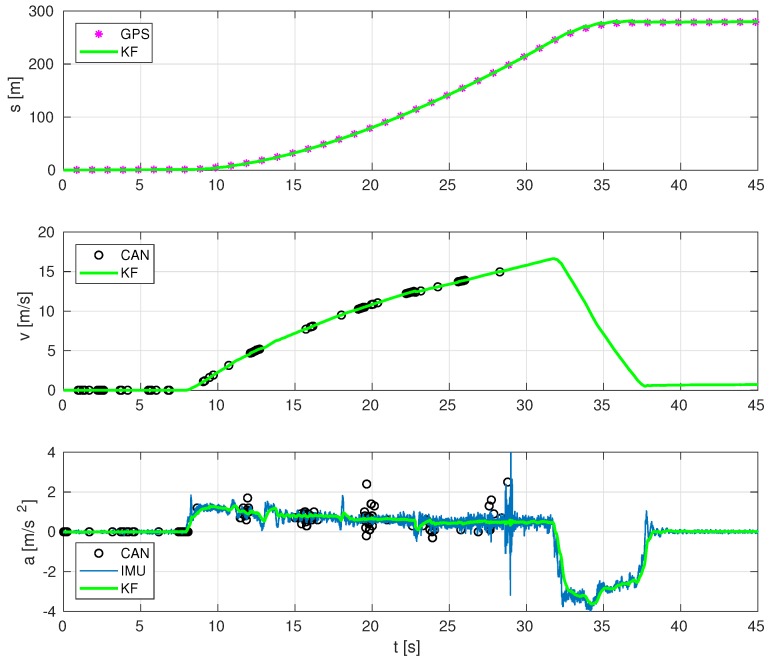
Estimation at a high rate of vehicle’s position, velocity and acceleration, for the test at 60 km/h and intense braking.

Figure 11 shows that our proposed sensor fusion works satisfactorily in an experiment driving up to 60 km/h and intense braking, and also the relevance of including CAN data. In this particular experiment, at a time of 28 s, CAN signal is lost. Before losing this signal, the estimation provided by our sensor fusion is reasonably good. However, when CAN signal is lost, IMU-GPS data provides acceptable position and acceleration estimates, as intuitively expected, but it is inaccurate in the speed estimation: an offset of approximately 1 m/s is present even with the bus being stopped (GPS readings are not constant), whereas, in the initial stages, as well as in Figure 9, such speed offset is not present when CAN data are available.

**Figure 12:**
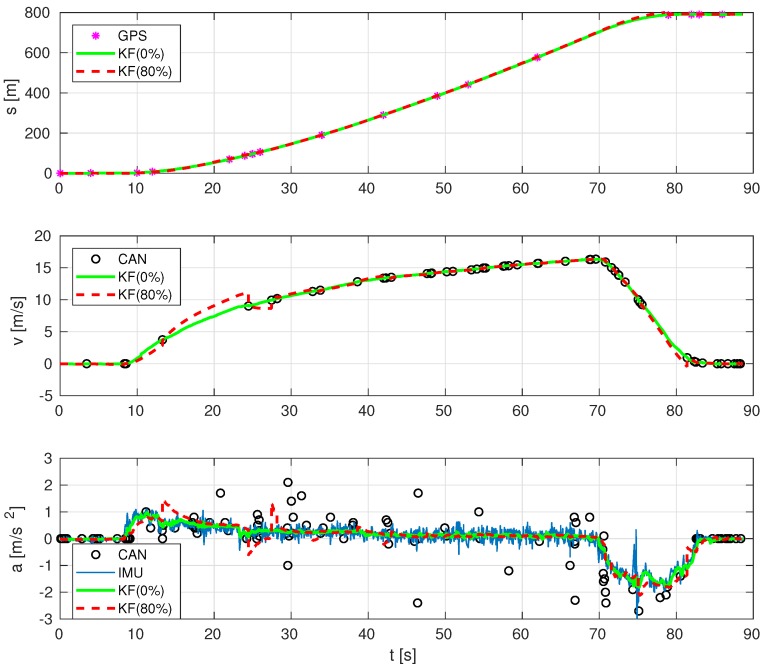
Estimation at a high rate of vehicle’s position, velocity and acceleration, with 80% of data missing.

**Figure 13:**
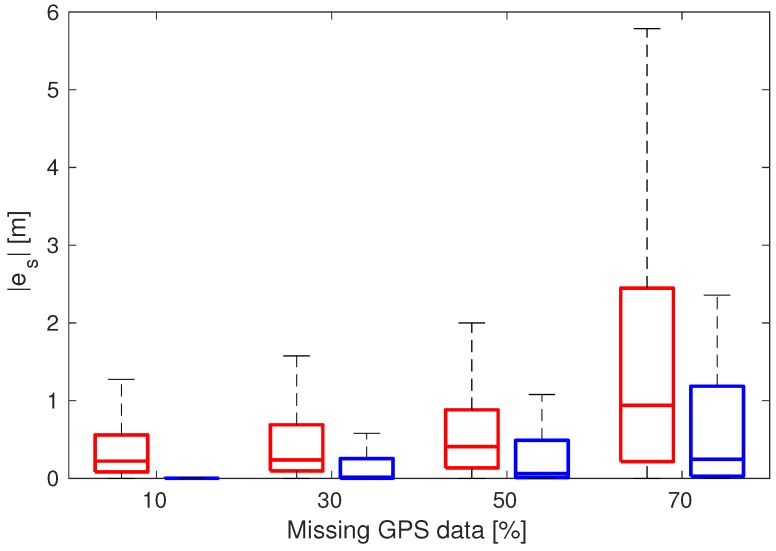
Absolute estimation error $|e_{s}|$ of the travelled distance *s* for different percentages of missing GPS data: without CAN (red boxes) and with CAN (blue boxes).

Next, Figure 12 shows the robustness of the resulting estimations with 80% of randomly missing measurements, which means that, among all available samplings, we have randomly removed up to 80% of data. The combination of GPS, CAN and IMU holds reasonably well despite having only 20% of the measurements compared to Figure 9, due to the high data correlation arising from our models. In addition to that, Figure 13 depicts the whisker plot of the absolute values of the difference between position estimates with 0% missing data and randomly missing GPS data in a given percentage. The red whiskers correspond to the case where CAN data are not used, while the blue whiskers use the same amount of GPS data, but include CAN measurements. The result shows that using CAN yields a much more robust estimate under missing GPS data conditions.

## 6. Discussion

The main contributions of this work are the design of an experimental setup for data collection in urban transport passenger buses, complying with non-invasive safety-related requirements, and the subsequent implementation of a sensor fusion methodology able to combine data coming from different sources at different, and possibly irregular, sampling rates. Throughout the paper, it has been shown that the proposed data fusion algorithm can estimate accurately the state variables of the vehicle at a high frequency, even for heterogeneous sensors irregularly sampled (with a common base period), and in cases where there are missing samples and data are sparse. The results also show that offsets from sensors and the model itself can be compensated, thanks to the use of the proposed methodology.

Another result is the fact that the estimation of kinematic and dynamic variables is considerably improved by using some information published by vehicle’s internal ECUs through its CAN network. This is essential for parameter identification, especially when the model is intended to be used in a simulator to accurately reproduce the behaviour of the vehicle. Furthermore, thanks to getting access to data from the vehicle’s internal sensors (engine torque and angular velocity, gear, transmission ratio, brake pressure, etc.), there is no need for extra sensorization, which is hard to mount on this kind of vehicle.

The results obtained in this paper are promising, and they can be used by specialised mechanical engineers in order to identify some parameters related to vehicle’s longitudinal dynamics. In this sense, the paper contributes as an important preliminary step for processing all the data necessary for a vehicle’s longitudinal dynamics identification, with the goal of our further work being to identify the main dynamic parameters of an urban bus, in order to use its model in a driving simulator (Figure 1a), in order to achieve a faithful replication of the driving conditions for a particular bus model. The work represented may be used, too, in many other applications that require robustness to irregular sampling and missing data where the outcome of the estimated values are of interest.

## Abbreviations

The following abbreviations are used in this manuscript:

CAN	Controller Area Network
ECU	Electronic Control Unit
FMS	Fleet Management System
GPS	Global Positioning System
IMU	Inertial Measurement Unit
ISO	International Organization for Standardization
KF	Kalman Filter
NMEA	National Marine Electronics Association
PGN	Parameter Group Number
RMS	Root Mean Square
RPM	Revolutions Per Minute
SAE	Society of Automotive Engineers
SPN	Suspect Parameter Numbers
LCM	Least Common Multiple

## Appendix A. J1939 Messages Used for Data Fusion

**Table A1:** Variables from CAN used.

PGN	Acronym	Label	SPNs
61440	ERC1	Electronic Retarder Controller 1	520 (Actual Retarder-Percent Torque)
61441	EBC1	Electronic Brake Controller 1	521 (Brake Pedal Position)
61442	ETC1	Electronic Transmission Controller 1	161 (Transmission Input Shaft Speed) 191 (Transmission Output Shaft Speed)
61443	EEC2	Electronic Engine Controller 2	91 (Accelerator Pedal Position 1) 92 (Engine Percent Load At Current Speed)
61444	EEC1	Electronic Engine Controller 1	190 (Engine Speed) 513 (Actual Engine-Percent Torque)
61445	ETC2	Electronic Transmission Controller 2	523 (Transmission Current Gear) 524 (Transmission Selected Gear) 526 (Transmission Actual Gear Ratio)
61449	VDC2	Vehicle Dynamic Stability Control 2	1807 (Steering wheel Angle) 1810 (Longitudinal Acceleration)
61452	ETC8	Electronic Transmission Controller #8	3030 (Transmission Torque Converter Ratio)
65132	TCO1	Tachograph	1623 (Tachograph output shaft speed) 1624 (Tachograph vehicle speed)
65215	EBC2	Wheel Speed Information	905 (Relative Speed; Front Axle, Left Wheel) 906 (Relative Speed; Front Axle, Right Wheel) 908 (Relative Speed; Rear Axle #1, Right Wheel)
65247	EEC3	Electronic Engine Controller 3	514 (Nominal Friction-Percent Torque)
65265	CCVS	Cruise Control/Vehicle Speed	84 (Wheel-Based Vehicle Speed)
